# Acetyl-CoA metabolism drives epigenome change and contributes to carcinogenesis risk in fatty liver disease

**DOI:** 10.1186/s13073-022-01071-5

**Published:** 2022-06-23

**Authors:** Gabriella Assante, Sriram Chandrasekaran, Stanley Ng, Aikaterini Tourna, Carolina H. Chung, Kowsar A. Isse, Jasmine L. Banks, Ugo Soffientini, Celine Filippi, Anil Dhawan, Mo Liu, Steven G. Rozen, Matthew Hoare, Peter Campbell, J. William O. Ballard, Nigel Turner, Margaret J. Morris, Shilpa Chokshi, Neil A. Youngson

**Affiliations:** 1 Institute of Hepatology, Foundation for Liver Research, 111 Coldharbour Lane, London, SE5 9NT UK; 2 King’s College London, Faculty of Life Sciences and Medicine, London, UK; 3 Program in Chemical Biology, University of Michigan, Ann Arbor, MI 48109 USA; 4 Center for Bioinformatics and Computational Medicine, Ann Arbor, MI 48109 USA; 5 Department of Biomedical Engineering, University of Michigan, Ann Arbor, MI 48109 USA; 6 Rogel Cancer Center, University of Michigan Medical School, Ann Arbor, MI 48109 USA; 7 Wellcome Trust Sanger Institute, Cambridge, UK; 8 UNSW Sydney, Sydney, Australia; 9 Cellular Bioenergetics Laboratory, Victor Chang Cardiac Research Institute, Darlinghurst, NSW Australia; 10 Institute of Liver Studies, King’s College Hospital, London, UK; 11 Programme in Cancer and Stem Cell Biology, Duke-NUS Medical School, Singapore, Singapore; 12 CRUK Cambridge Institute, Cambridge, UK; 13 Department of Medicine, University of Cambridge, Addenbrooke’s Hospital, Cambridge, UK; 14 Department of Ecology, Environment and Evolution, La Trobe University, Bundoora, Melbourne, VIC 3086 Australia

**Keywords:** Steatosis, Histone acetylation, Hepatocellular carcinoma, NAFLD, ARLD, Telomerase

## Abstract

**Background:**

The incidence of non-alcoholic fatty liver disease (NAFLD)-associated hepatocellular carcinoma (HCC) is increasing worldwide, but the steps in precancerous hepatocytes which lead to HCC driver mutations are not well understood. Here we provide evidence that metabolically driven histone hyperacetylation in steatotic hepatocytes can increase DNA damage to initiate carcinogenesis.

**Methods:**

Global epigenetic state was assessed in liver samples from high-fat diet or high-fructose diet rodent models, as well as in cultured immortalized human hepatocytes (IHH cells). The mechanisms linking steatosis, histone acetylation and DNA damage were investigated by computational metabolic modelling as well as through manipulation of IHH cells with metabolic and epigenetic inhibitors. Chromatin immunoprecipitation and next-generation sequencing (ChIP-seq) and transcriptome (RNA-seq) analyses were performed on IHH cells. Mutation locations and patterns were compared between the IHH cell model and genome sequence data from preneoplastic fatty liver samples from patients with alcohol-related liver disease and NAFLD.

**Results:**

Genome-wide histone acetylation was increased in steatotic livers of rodents fed high-fructose or high-fat diet. In vitro, steatosis relaxed chromatin and increased DNA damage marker γH2AX, which was reversed by inhibiting acetyl-CoA production. Steatosis-associated acetylation and γH2AX were enriched at gene clusters in telomere-proximal regions which contained HCC tumour suppressors in hepatocytes and human fatty livers. Regions of metabolically driven epigenetic change also had increased levels of DNA mutation in non-cancerous tissue from NAFLD and alcohol-related liver disease patients. Finally, genome-scale network modelling indicated that redox balance could be a key contributor to this mechanism.

**Conclusions:**

Abnormal histone hyperacetylation facilitates DNA damage in steatotic hepatocytes and is a potential initiating event in hepatocellular carcinogenesis.

**Supplementary Information:**

The online version contains supplementary material available at 10.1186/s13073-022-01071-5.

## Background

Hepatocellular carcinoma (HCC) is the most common type of primary liver cancer and the fourth most common cause of cancer-related death worldwide [1]. Its incidence is increasing across the globe, a major contributor to this being the obesity epidemic and the concomitant increases in non-alcoholic fatty liver disease (NAFLD). At present NAFLD is estimated to affect around 25% of the world’s population [2] and is the fastest growing cause of HCC in the USA, France and the UK [3]. Clinically, NAFLD presents as a spectrum which progresses from simple steatosis to non-alcoholic steatohepatitis (NASH) with accumulating inflammation and fibrosis culminating in cirrhosis and eventually liver failure. While HCC risk is highest at the more severe stages, there is still increased risk in fatty livers without cirrhosis [4, 5]. HCC is a heterogeneous cancer and can display at least 6 subtypes, each with a different combination of characteristic mutations and transcriptomes [6]. The most common genetic signature of HCC is mutation of the *Telomerase* (*TERT*) gene which is present in around 60% of cancers and is the earliest detectable of all the known mutations [7]. HCC-associated *TERT* mutations activate the gene, preventing telomere shortening which subverts cellular senescence and apoptosis programmes thus setting cells on the path for immortalisation [7, 8]. The underlying causes for *TERT* mutation can include viral (hepatitis B or C) insertion, point mutations which alter localised transcription factor binding, or larger rearrangements which duplicate the gene or translocate the regulatory sequences of a more highly expressed gene to the *TERT* locus [7]. However, the mutational forces which induce NAFLD-HCC are poorly understood. Oxidative stress is a strong candidate for generating mutations [9] but the reasons why *TERT* mutations are so much more prevalent than other mutations at the early stages of carcinogenesis is unknown.

Diet and epigenetics are highly interlinked as the molecular substrates for epigenetic modifications are also products of intermediary metabolism [10]. This situation ensures that there is constant communication between the two processes, so that gene expression can be quickly modified to meet the energy demands of the cell. Perhaps the best understood example of the link between dietary macronutrients and epigenetics is histone acetylation. Acetyl coenzyme A (acetyl-CoA) is produced by the breakdown of carbohydrates [11, 12] and fats [13] for cellular energy, as well as being the substrate for de novo lipogenesis (DNL) and ketone generation. It also provides the acetyl groups for histone acetylation, an epigenetic modification which can decondense, i.e. ‘open-up’ chromatin to facilitate access to DNA for transcriptional or DNA repair proteins. In vivo measurements in rat have shown that absolute acetyl-CoA levels are increased in fatty liver [14]. In human NAFLD livers, acetyl-CoA flux into DNL, and oxidation in the tricarboxylic acid (TCA) cycle, have been shown to be upregulated, while ketogenesis is reduced compared to healthy liver [15].

The importance of epigenetic changes in cancer progression is well established, particularly in the context of changes which alter the transcription of oncogenes and tumour suppressor genes [16]. Mutations in epigenetic modifier proteins are commonly found in cancer, even as the initiating mutation [17, 18]. The potential therapeutic opportunity of this relationship is currently being tested in the context of histone acetylation with several clinical trials of pharmacological inhibitors of the writers, histone acetyltransferases (HATs) and erasers, histone deacetylases (HDACs) in a variety of cancers, including HCC [19, 20]. These therapies aim to reverse epigenetic dysregulation in partially or fully developed cancers, *after* cancer-promoting DNA mutations have occurred, rather than at the initial pre-mutational stages that promote carcinogenesis. This restricted use of epigenetic therapies is partly due to there being no previously reported examples of abnormal energy metabolism driving epigenetic dysregulation to initiate carcinogenesis. Considering the mechanistic links between metabolism, epigenetics and cancer, we hypothesized that perturbed acetyl-CoA metabolism and histone acetylation could be an unrecognized contributor to NAFLD pathology and HCC risk. We investigated this using cell culture and rodent models of hepatocyte steatosis, as well as a computational model linking cellular metabolism and histone acetylation. To support the existence of a common pathological mechanism in humans, data from the in vitro model was compared with mutational data from human preneoplastic livers and HCC samples.

## Methods

### Animal work

All rodent experiments were approved by the University of New South Wales Animal Care and Ethics Committee (Project numbers ACEC 11/82B and 13/55B). For the high-fat diet treatment (HFD), male Sprague–Dawley rats from the Animal Research Centre (ARC, Perth, Australia) were housed two per cage under a 12:12 h light/dark cycle and ad libitum access to water and experimental diets. Three-week-old rats were split into two groups with equal average body weight (*n* = 14/14). Control rats were fed normal chow (energy: 11 kJ g^−1^, 12% fat, 21% protein, 65% carbohydrate; Gordon’s Stockfeeds, NSW, Australia) while the HFD group was provided with two commercial HFD pellets, SF03-020 (20 kJ g^−1^, 43% fat, 17% protein, 40% carbohydrate; Specialty feeds, Glen Forest, WA, Australia) and SF01-025 (18.3 kJ g^−1^, 44% fat, 17% protein, 39% carbohydrate; Specialty feeds), as well as normal chow. The rats were sacrificed between 24 and 29 weeks of age. Animals were sacrificed after anaesthesia induced by i.p. injection of 100 mg ketamine/kg body weight and 15 mg xylazine/kg body weight followed by decapitation. At sacrifice the difference in body weight between the groups had increased so that the control group averaged ± s.d 535 ± 67 g (range 451–631 g) and the HFD group 717 ± 67 g (range 604–807 g), *P* = 6.0 × 10^−8^ [21].

For high-fructose diet (HF), 10-week-old C57BL/6 J mice were purchased from the Australian Resource Centre (Perth, Australia). Mice were maintained in a temperature-controlled room (22 °C ± 1 °C) with a 12-h light/dark cycle and ad libitum access to water and experimental diets. After 1 week on a standard control ‘chow’ diet (71% of calories from carbohydrate as wheat/starch, 8% calories from fat, 21% calories from protein, ~ 3 kcal/g; Gordon’s Specialty Stock Feeds, NSW, Australia), mice were randomly allocated to remain on the chow diet (C) or to receive a home-made diet enriched in fructose (FR; 35% of calories from fructose, 35% calories from starch, 10% calories from fat, 20% calories from protein, ~ 3.1 kcal/g) ad libitum for 8 weeks. At sacrifice, the control and fructose-fed mice had no body weight difference but liver triacylglycerol levels in fructose mice were 225% the level of control mice *p* < 0.001 [22].

### Western blots (immunoblotting)

Histones were extracted from approximately 30 mg powdered liver on a Precellys (Sapphire Bioscience Australia) at 6 m/s for 30 s, in Triton Extraction Buffer (TEB: PBS containing 0.5% Triton X 100 (v/v), sodium butyrate 5 mM) using the Abcam Acid Extraction Histone extraction protocol for western blot. Briefly, after lysis, samples were centrifuged at 6500 × *g* for 10 min at 4 °C to spin down the nuclei and supernatant discarded; the pellet is resuspended in 0.2 N HCl, and histones are acid extracted overnight at 4 °C on a rotator. The next-day samples were centrifuged at 6500 × *g* for 10 min at 4 °C to pellet debris. The supernatant (which contains histones) was neutralized with 2 M NaOH at 1/10 of supernatant volume. Equal amounts of protein lysate were electrophoresed through a 4–15% precast gel (Criterion TGX, Bio-Rad) for 45 min at 150 V in running buffer (25 mmol/l Tris base, 192 mmol/l glycine and 1% SDS, pH 8.3). Proteins were transferred via a semi dry transfer process with a Trans Blot Turbo System (Bio-Rad) onto PVDF membranes (Bio-Rad). Membranes were blocked in 4% BSA in TBS-Tween for 1 h, then incubated overnight at 4 °C with primary antibodies used at 1:2000 dilution; Histone H4K16ac pAb (Active Motif 39167), total histone H3 (Abcam, ab1791), Histone H2A.X antibody #2595 (Cell Signaling) and Phospho-Histone H2A.X (Ser139) (20E3) Rabbit mAb #9718 (Cell Signaling). The membrane was subjected to three 10-min washes with TBS-Tween and incubation with appropriate secondary antibody (Cell Signaling) in 2% skim milk blocking solution in TBS-Tween at room temperature for 1 h, followed by three 10-min washes with TBS-Tween. For detecting bands, membranes were exposed to Clarity Western ECL Substrate (Bio-Rad) and visualized on a Bio-Rad ChemiDoc XRS. Membranes were stripped using Reblot Plus (10X) (Millipore) for 10 min at room temperature and were re-blocked prior to pan-H3 immunoblotting. Immunolabelled bands were quantitated using ImageJ 1.44p software.

### Cell culture

Immortalized human hepatocytes (IHH) were cultured under standard conditions using control media DMEM/F-12 (Sigma) without phenol red, 10% FBS (Life Technologies, 10,108–165), 1% PEN/STREP (Life Technologies 15,140–122), 0.1% L-Glutamine (Life Technologies 25,030–024), 0.02% dexamethasone (Sigma D49025MG) and 1 pM insulin human recombinant zinc (Life Technologies 12,585–014). For generating steatosis, cells were supplemented with oleic acid-albumin (Sigma O3008) (300 μM). After for 4 days, confluent cells were treated with 200 μM etomoxir (Sigma E1905), 50 μM BMS 303,141 (Sigma SML0784), 5 μM garcinol (Thermo Fisher 15,716,585), acetyl-CoA carboxylase (ACC) inhibitor firsocostat (GS-0976) (Selleckchem S8893) 50 nM, antioxidant supplement (Sigma A1345) 10 × , methotrexate (Sigma M9929) 10 nM and 50 μM ACCSi—CAS 508186–14-9 (Sigma 5,337,560,001) for 4 h at 37 °C.

### Lipid droplets staining

Intracellular lipid droplet staining was performed with HCS LipidTOX™ Green Neutral Lipid Stain (Thermo Fisher). After removing the incubation medium, the cells were fixed with a solution of 3.7% of formaldehyde (Sigma) supplemented with Hoechst 33342 (Thermo Fisher) and incubated for 20 min at room temperature in the dark. After aspirating the fixative solution, the cells were washed twice with PBS without Ca/Mg (Life Technologies). LipidTOX™ neutral lipid stain diluted in PBS was added to the cells and incubated for 1 h at room temperature in the dark at a final concentration of 1 × . Cell imaging acquisition was performed by using GFP 488 nm and DAPI 355 nm on Cytation 5 Cell Imaging Multi-Mode Reader (BioTek).

### In situ DNAse I sensitivity assay: chromatin state

CSK buffer was made by dissolving Pipes/KOH (Sigma), NaCl (Sigma), Sucrose (Sigma), EGTA (Sigma) and MgCl_2_ (Sigma) in H_2_O. Cover slides were coated for 1 h with 20% w/v of fibronectin (Sigma) diluted in PBS (Life Technology) at room temperature. Next, cells were plated on the coated cover slides, washed once with PBS and lysed in CSK buffer supplemented with 0.2% Triton X-100 (Sigma) and cOmplete™ Protease Inhibitor Cocktail (Sigma/Roche) for 5 min at room temperature. Once the incubated solution was removed, the cells were washed with CSK buffer and then were incubated in CSK buffer supplemented with 0.1% Triton X-100 (Sigma), cOmplete™ Protease Inhibitor Cocktail (Sigma/Roche) and 50 U/ml DNaseI (Sigma) for 20 min at room temperature. Cells were then washed with CSK buffer, and the remaining DNA was stained using Hoechst 33342 (Thermo Fisher) at a concentration of 5 μg/ml in CSK buffer supplemented with 125 mM ammonium sulfate (Sigma) and cOmplete™ Protease Inhibitor Cocktail (Sigma/Roche) for 5 min at room temperature. Cells were washed in CSK buffer and fixed in 100% methanol (Sigma) for 5 min at − 20 °C. The methanol was removed and the CSK buffer was added before imaging nucleus acquisition by using DAPI 355 nm on Cytation 5 Cell Imaging Multi-Mode Reader (BioTek). The nucleus area of cells was acquired by a mask function created on the Microscope Software.

### Indirect ɣH2AX immunofluorescence

Control and steatotic cells were washed twice in PBS (Life Technology) after 4 h drug treatment. Next, cells were fixed with 4% solution of formaldehyde (Sigma) for 15 min at room temperature and washed twice in PBS. Then, cells were permeabilized with 0.25% Triton X-100 (Sigma) for 10 min at room temperature, washed twice in PBS and incubated with 3% BSA (Sigma) for 1 h at room temperature. Cells were first incubated with anti-gamma H2A.X (phospho S139) antibody—ChIP Grade (Abcam) for 1 h at 37 °C and then incubated with fluorochrome-conjugated secondary antibody (Sigma SAB4600400) for 1 h at 37 °C in the dark. Cells were then stained with 1 μg/mL Hoechst 33342 (Thermo Fisher) for 10 min at room temperature and the imaging acquisition was performed by using DAPI 355 nm and RFP 558 nm on Cytation 5 Cell Imaging Multi-Mode Reader (BioTek). The percentage of positive cells was calculated with a mask function created on the Microscope Software.

### ChIP and next-generation sequencing

Chromatin was isolated from 20 million cells using the Magna ChIP A/G Kit (One-day chromatin Immunoprecipitation Kits, Merck) according to the manufacturer’s instructions. Chromatin was sonicated with a Vibra-Cell VCX750 probe sonicator (Sonics) for 3 pulses of 15 s separated by 30-s intervals at 30% output. Fifty microliters of chromatin was immunoprecipitated with 5 μg antibody specific for anti-gamma H2A.X (phospho S139) antibody—ChIP Grade (Abcam) or with 5 μg antibody specific for histone H4K16ac (ActiveMotif). In total, 100–250 ng of isolated DNA (average size 500 bp) was sent to Genewiz for sequencing in an Illumina HiSeq 2 × 150 bp. Sequencing yield was 29–37 Mb per sample. Sequence analysis was also performed by Genewiz. Raw data are available to download [23]. Sequence reads were trimmed to remove possible adapter sequences and nucleotides with poor quality at 3 end (error rate > 0.01) using CLC Genomics Server 9.0. Trimmed data was then aligned to reference genome for human genome hg38. During the mapping, only specific alignment was allowed. A total of 163–198 M reads were aligned (all > 95%). Peak analysis for each sample was done using the Histone model algorithm. As a result, a list of peaks (*p* < 0.05) was obtained from each treatment sample. Detected peak sequences were extracted and peak coverages were calculated. Total peak number from control media samples were 2815 and 1291 from the H4K16ac and ɣH2AX ChIP-seqs, respectively. Total peak number from oleic acid-treated samples were 650 and 5912 from the H4K16ac and ɣH2AX ChIP-seqs, respectively.

### ChIP and quantitative PCR on cells and human livers

Samples of human livers which had been rejected for transplantation (1 healthy liver and 1 steatotic liver) were acquired through the King’s College Hospital, London; ethics number for hepatocyte biology held by Professor Dhawan and Dr Filippi is LREC protocol 1998–0249. Chromatin was isolated from 20 million IHH cells or 40 mg of human fatty liver (using the EpiQuik Chromatin Immunoprecipitation Kit (Epigentek) according to the manufacturer’s instructions for cells or tissues. Sonication on isolated chromatin was performed as for the ChIP-seq for cells but with pulses increased to 4 for tissue. Real-time PCRs were performed in triplicate with primers for *TERT* promoter, PTEN and TP53 by using Luna® Universal qPCR Master Mix (NEB – M0003) as SybrGreen Probe on an Ariamx Real-Time PCR System (Agilent). Primer sequences for qPCR were as follows: PTEN ChIP-qPCR Forward 5′-*GAGTCGCCTGTCACCATTTC-3*′PTEN ChIP-qPCR *Reverse 5*′*-GCGCACGGGAGGTTTAAAA-3*′; TERTp ChIP-qPCR *Forward 5*′*-GGATTCGCGGGCACAGAC-3*′, TERTp ChIP-qPCR *Reverse 5*′*-AGCGCTGCCTGAAACTCG-3*′; TP53 ChIP-qPCR *Forward 5*′*-GTACCACCATCCACTACAACTACATGT-3*′, TP53 ChIP-qPCR *Reverse 5*′*-GGCTCCTGACCTGGAGTCTTC-3*′*.*

### RNA sequencing

IHH cells were grown in either control media, oleic acid-supplemented media or oleic acid-supplemented media with a 4-h treatment of the histone acetyltransferase inhibitor (HATi) garcinol prior to collection. Four separate replicates were performed for each group. Total RNA was isolated from 8 million cells per sample using a Qiagen RNEasy Mini kit. Approximately 1 μg of RNA was sent to Novogene UK for directional library preparation (with rRNA removal), Illumina NovaSeq PE150 sequencing and bioinformatic analysis. Sequencing yield was 5–6 Gb per sample. Sequence reads were trimmed to remove adapter sequences, reads with uncertain nucleotides constituting more than 10%, or reads which had low-quality nucleotides (Base Quality less than 5) constituting more than 50% of the read. Trimmed data were then aligned to the reference human genome hg38 with HISAT2 [24]. Aligned read BAM files are available to download [23]. In total, 28–49 M reads uniquely aligned from each sample (85–95% of all reads). Mapping information from all samples was combined and placed as input into a Cufflinks assembler. Assembled transfrags were then compared to the reference transcripts to identify known and novel genes. Differential gene expression analysis between groups was performed with DEseq2 [25]. GATK software was used to perform mutation site analysis on sample data and Snpeff software used to annotate the variant [26]. Gene enrichment analysis was performed on significantly differentially expressed genes at http://www.geneontology.org/ and https://david.ncifcrf.gov/.

### Statistical analysis for wet-lab work

Results are expressed as mean ± SEM. Data were analysed using Student’s *t* test or one-way ANOVA, followed by post hoc LSD tests using GraphPad Prism.

### Mutation burden comparisons

The ethics number under which the samples were acquired for mutational burden comparisons and mutational signature extraction was 16/NI/0196 as approved by the Cambridge University Hospitals NHS Foundation Trust. All biological samples were collected with informed consent from Addenbrooke’s Hospital, Cambridge, UK. Comparisons between the number of SNVs detected in a whole genome sequencing (WGS) dataset derived from human liver biopsies [26] between various clinically annotated conditions were performed in the R statistical programming using the ggstatsplot package [27]. For all comparisons, the default non-parametric test was used as defined by the software, while the Benjamini–Hochberg method was enabled to correct *p*-values for multiple hypothesis testing. The normal liver donors were 5 colorectal cancer patients with average Kleiner Fibrosis Scores of 0.8 and average BMI of 26.6, 19 NAFLD patients with an average Kleiner Fibrosis Score of 3.2 and average BMI of 30.8 and 10 ARLD patients with an average Kleiner Fibrosis Score of 3.9 and average BMI of 26.1 (Additional file 1: Liver Cohort Baseline Clinical Data).

### Mutational signature extraction

Using the same files as for the mutation burden comparisons, single base substitution signatures in 96-trinucleotide contexts was extracted using the mSigHdp (v1.1.2) and hdpx (v0.3.0) package in R. In total, 50,000 burn-in iterations were used, while setting the following hyperparameters and posterior sampling variables: post.n = 200, post.space = 100, gamma.alpha = 1, gamma.beta = 20. All other parameters were set to defaults. Cosine similarity was calculated to compare the extracted mutational signatures to the compendium of single base substitution COSMIC signatures [28].

### Computational metabolic modelling

Genome-scale metabolic models have been widely used to predict the metabolic behaviour of various mammalian cell types using transcriptomics data [29, 30]. Gene expression data from mouse hepatocyte AML12 cells treated with fatty acids (Octanoate) from McDonnel et al. [13] was used as input to derive reaction flux through the human genome-scale metabolic model (RECON1) [31]. The human orthologs of the lists of up- and downregulated mouse genes were overlaid onto the RECON1 model based on gene-protein-reaction annotations in the model. Reaction fluxes that best fit the expression data while satisfying stoichiometric and thermodynamic constraints were determined using a modelling approach detailed in Shen et al. [32, 33]. This approach maximizes flux through reactions that are upregulated while minimizing flux through those reactions that are downregulated using linear optimization. The exchange reactions for nutrients (i.e. glucose, amino acids, fatty acids, vitamins and minerals) in the metabolic model were constrained based on media composition used in this study (DMEM-F12 media with 500 μM oleic acid). For visualization, a *z*-score transformation was performed on the flux difference between control and treatment groups, and the significance of the difference was determined using a paired *t*-test. Reactions showing significant changes in flux (*p*-value < 0.05) were visualized in the heatmap (excluding transport-, exchange- and pseudo- reactions).

## Results

### Histone acetylation and chromatin state is altered in liver steatosis

**Fig. 1:**
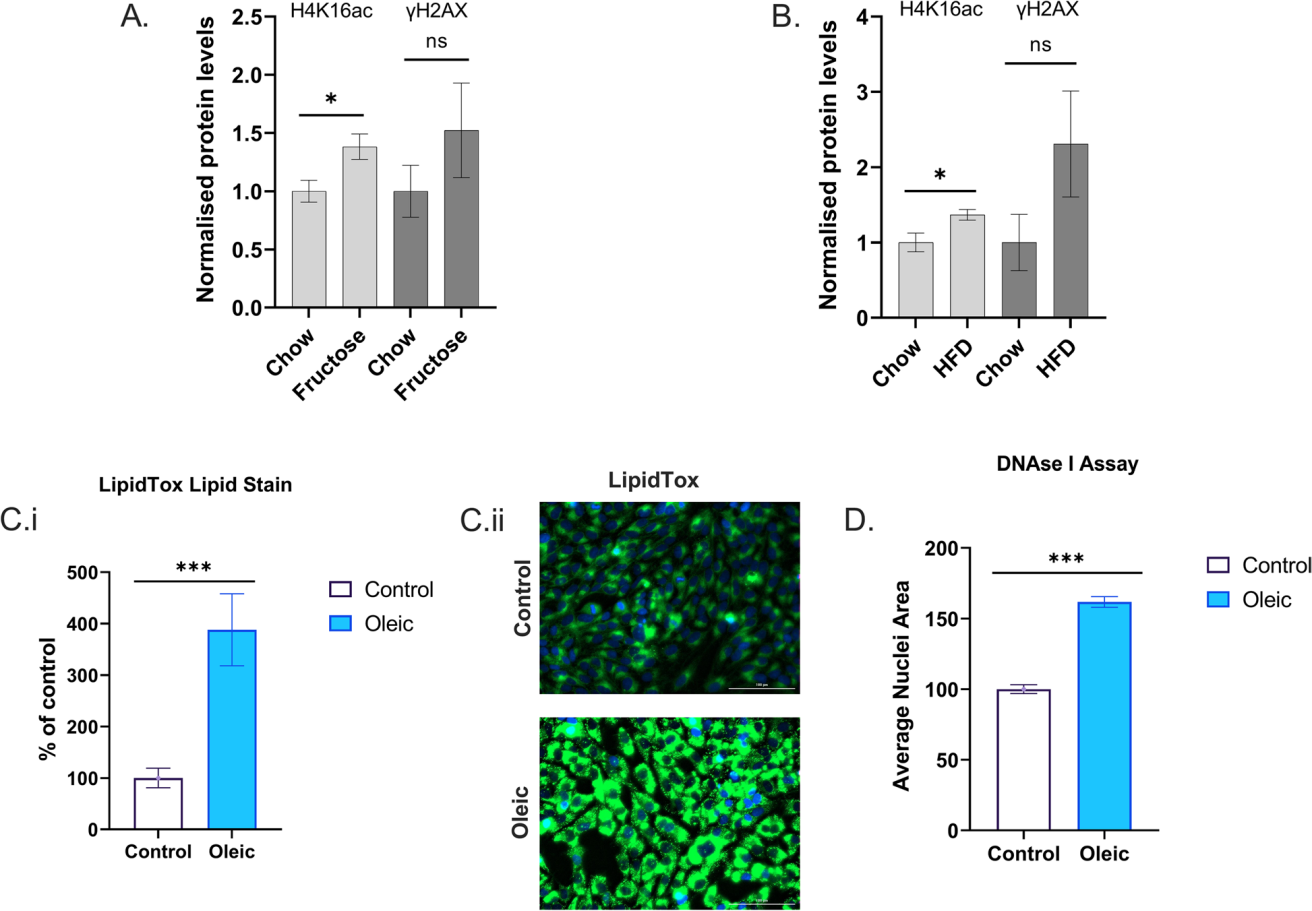
**A** Histone acetylation and γH2AX levels in livers of control and high-fructose diet mice. **B** Histone acetylation and γH2AX levels in livers of control and high-fat diet rats. **Ci** Lipid content of IHH cells cultured in control or oleic acid-supplemented media. **Cii** Representative fluorescence microscopy image of IHH cells cultured in control or oleic acid-supplemented media (blue nuclear stain and green lipid stain). **D** Nuclear area measurement of IHH cells cultured in control or oleic acid-supplemented media after DNAse I treatment. Error bars are ± SEM. Histone westerns all groups *n* = 4, Lipidtox assay *n* = 7/8, DNaseI Assay *n* = 3. *P < 0.05, ***P < 0.001 in *t*-test

To test the hypothesis that histone acetylation levels are increased in steatotic hepatocytes, we first examined rodent and cell culture models of liver and hepatocyte steatosis. Using our established rodent models of NAFLD, we found in both the mouse model of fructose diet-induced liver steatosis (Fig. 1A) [22] and the rat model of high-fat diet-induced steatosis (Fig. 1B) [21], an increased H4K16 acetylation compared to control diet animals. To dissect the mechanisms that link macronutrients and genome-wide epigenetic state, we treated immortalized human hepatocytes (IHH) with oleic acid for 4 days. Oleic acid is the most common fatty acid in nature and is abundant in dietary fats from plant or animal sources. This treatment quadrupled intracellular lipid content (Fig. 1 Ci–ii). As a further test for broad epigenomic change, we adapted a method for assessing genome-wide nuclear condensation in these cells [34] and found that oleic acid treatment significantly decondensed chromatin compared to cells grown in control media (Fig. 1D). These results supported our hypothesis that steatosis is associated with extensive histone acetylation and epigenomic change. The clinical importance of steatosis-associated epigenomic change per se could be limited as there are a variety of effective methods for reduction of steatosis (such as dieting, bariatric surgery and anti-lipogenic drugs). However, we also hypothesized that the ‘opening-up’ of chromatin could increase the exposure of DNA to mutagens such as reactive oxygen species (ROS), and DNA mutations could persist even after reversal of steatosis. In that case, the epigenomic change could be an indirect, yet clinically significant contributor to long-term liver damage and carcinogenesis in hepatocytes.

### Hepatocyte steatosis is associated with increased ɣH2AX which is reversed by inhibition of acetyl-CoA production

**Fig. 2:**
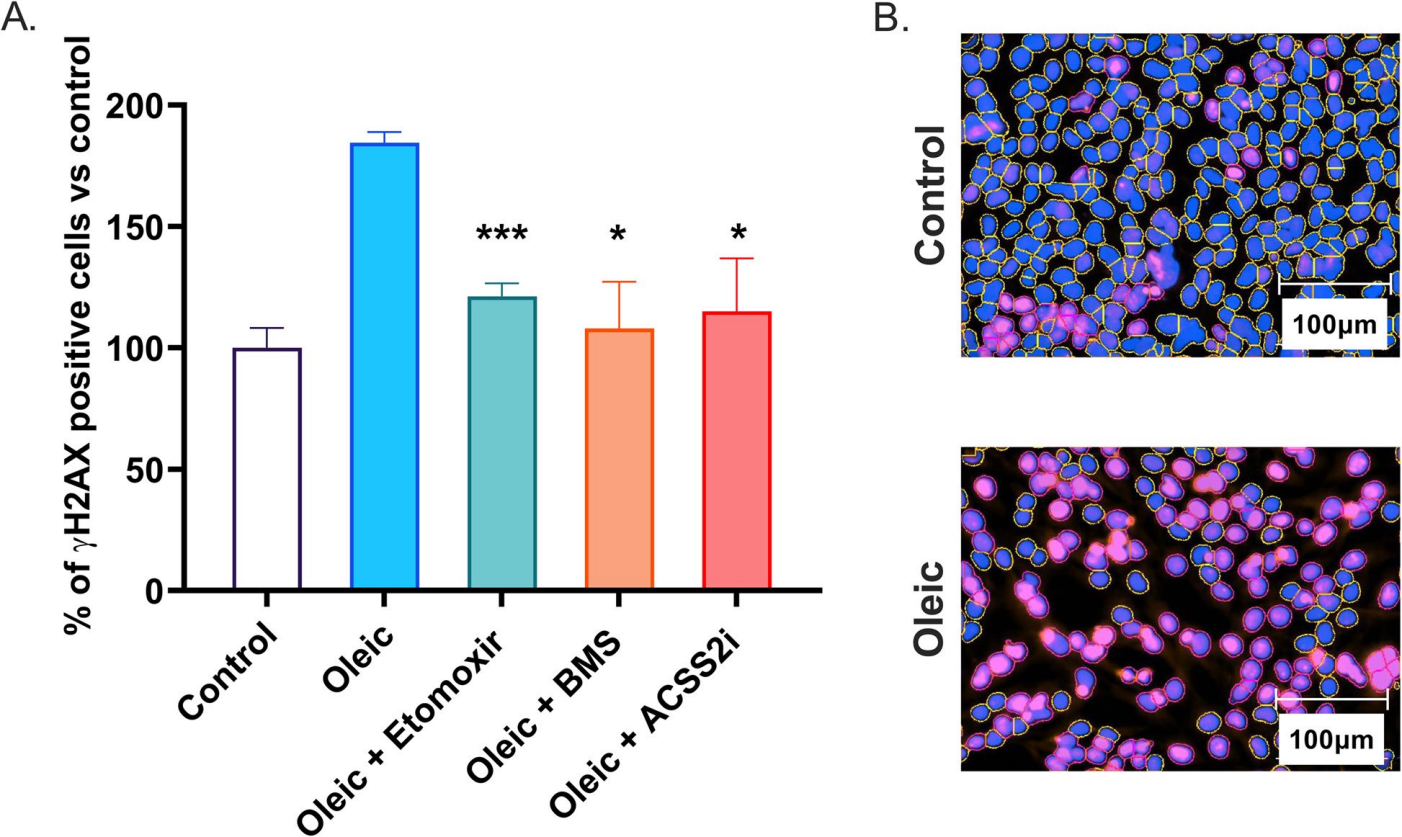
**A** Average number of cells with high ɣH2AX after oleic acid or oleic acid plus metabolic inhibitor drug treatments (% compared to control cells). **B** Representative ɣH2AX immunofluorescence images of control and oleic acid-treated cells (blue nuclear stain, pink ɣH2AX). Error bars are ± SEM. All groups *n* = 6–8. *P < 0.05, ***P < 0.0001 in *t*-test of oleic vs oleic plus inhibitor groups

We assessed whether the global chromatin changes are associated with DNA damage by performing immunofluorescence for ɣH2AX, a well-established marker of DNA damage [35, 36] which is present at elevated levels in steatotic liver [37, 38]. Oleic acid treatment nearly doubled the percentage of IHH cells which had high levels of ɣH2AX, compared to control media cells (Fig. 2A, B). A trend for increased γH2AX was also seen in the rodent livers which had steatosis and increased histone acetylation (Fig. 1A, B).

To investigate whether the DNA damage in vitro was dependent on histone acetylation, we inhibited the major biochemical pathways which produce acetyl-CoA, the substrate for histone acetylation. We found that the steatosis-associated ɣH2AX was reversed by a 4-h pharmacological inhibition of any of the major sources of acetyl-CoA (Fig. 2A, B). β-oxidation produces acetyl-CoA through the sequential breakdown of fatty acids. We reduced β-oxidation with the carnitine palmitoyltransferase-1 (CPT-1) inhibitor etomoxir. The ATP citrate lyase (ACLY) inhibitor (BMS-303141) prevents the production of acetyl-CoA from citrate, while acetyl-CoA synthetase (ACSS2) inhibition prevents production of acetyl-CoA from acetate. Inhibition of all these sources of acetyl-CoA had the similar effect of reducing the number of cells with high ɣH2AX to levels comparable with non-steatotic cells. These data suggest that diet-induced acetyl-CoA levels are indeed associated with DNA damage in steatotic hepatocytes.

### Genomic regions with highest increases of ɣH2AX levels in steatotic hepatocytes are clustered and contain genes commonly mutated in HCC

We next sought to identify which regions of the genome experience steatosis-associated increased ɣH2AX as that would identify which genes and biological processes could be disrupted by DNA damage. We performed chromatin immunoprecipitation with anti-H4K16ac and anti-ɣH2AX antibodies and next-generation sequencing on the enriched DNA from control and steatotic IHH cells. The ɣH2AX peak regions were compared between the control and steatotic cells. Seventy-seven of the 100 regions with biggest peak increases in steatosis compared to control cells were clustered on 7 chromosomes (5, 7, 11, 15, 19, Y and the mitochondrial genome). These clusters displayed colocalization of H4K16 acetylation and ɣH2AX peaks (Additional file 2: Fig S1, Additional file 3: ChIP-seq significant histone peaks). Two clusters stood out due to their telomere-proximal location and the genes within them. We searched The Cancer Genome Atlas for evidence that mutations have previously been found in these clusters in HCC. The Liver Hepatocellular Carcinoma Case Set (Project ID TCGA-LIHC) has information from 377 patients. Thirty of the 32 protein-coding genes in the chromosome 19 cluster of genes had identified mutations (most frequently at *BRSK1*, *ZNF135* and *EPS8L1* and *NLRP2*) in the patients; similarly, 17 out of 21 genes at the chromosome 5 cluster had identified mutations (most frequently in *TERT*, *PLEGHG4B* and *IRX4*). However, closer examination of the carcinoma risk factors in the TCGA patient history showed no statistically significant difference in the frequency of mutations within these 2 clusters between a group of alcoholic liver disease plus NAFLD cases (69 plus 11, respectively) and a viral hepatitis group (78 hepatitis B, 32 hepatitis C cases). Nonetheless, the combination of the cluster-wide epigenetic changes and the mutations in multiple genes within these clusters supports the possibility that large chromosomal regions have increased mutation risk from metabolism-associated epigenetic change in steatosis, which may overlap with mutagenic processes in viral hepatitis. Furthermore, the observation that the *TERT* gene, which is the most commonly mutated gene in HCC, had high histone acetylation and ɣH2AX levels in steatotic cells suggested that the mechanism could contribute to carcinogenesis.

### Genomic regions with highest increases of ɣH2AX levels in steatotic hepatocytes have increased mutations in livers from NAFLD and alcohol-related liver disease patients

**Fig. 3:**
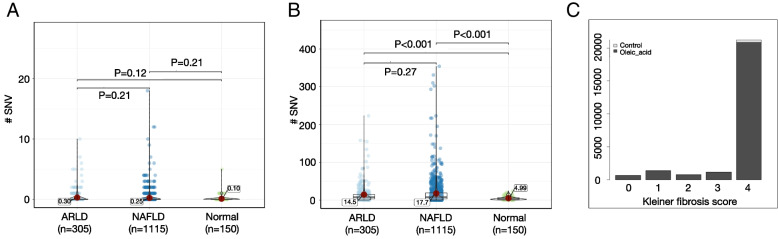
Violin plots showing a comparison of the number of single-nucleotide variants (SNVs) in whole genome sequencing from laser capture microdissections of human liver from patients with normal liver (*n* = 5), alcohol-related liver disease (ARLD) (*n* = 10) or non-alcoholic fatty liver disease (NAFLD) (*n* = 19), at **A** control and **B** oleic acid peak regions. The median number of SNVs are annotated on the plots. **C** Stratification of human liver disease cohort by Kleiner fibrosis classification. Significantly more base substitutions are present at oleic acid than control peak regions in patients at each stage of fibrosis (*p* < 0.001, Wilcoxon rank-sum test), with most SNVs in both groups in Kleiner score 4 livers

As the previous experiments were performed in cell culture and animal models, it was important to next investigate whether similar mechanisms occur in human. For this, we compared the ChIP-seq data with genomic mutations in human liver biopsies from chronic liver disease patients. In a previous publication, we described the sites of DNA mutation in cirrhotic livers with differing underlying aetiologies [39]. Here we compared the number of single-nucleotide variants (SNVs, i.e. mutations) detected in these whole genome sequencing (WGS) datasets derived from human liver biopsies, with the ɣH2AX peak regions in control media and oleic acid-treated IHH cells. These comparisons confirmed that the regions with high ɣH2AX levels in steatotic IHH cells were enriched in NAFLD and alcohol-related liver disease (ARLD)-associated SNVs (Fig. 3B), whereas the regions with high ɣH2AX levels in control media IHH cells were not (Fig. 3A). To examine the effect of fibrosis stage on point mutations, we stratified our human cohort according to the Kleiner fibrosis score. We then counted number of SNVs in each category and observed that there were significantly more base substitutions at oleic acid than control peak regions at each stage of fibrosis (*p* < 0.001, Wilcoxon rank-sum test, Fig. 3C), with most SNVs in both groups in Kleiner score 4 livers. Furthermore, a similar significant association was found between steatotic IHH ɣH2AX peak regions and SNVs in cirrhotic livers of type 2 diabetes patients, but not with the control media ɣH2AX peak regions (Additional file 2: Fig S2). These analyses suggest that the genomic regions which were identified to be sensitive to metabolism-associated epigenetic change in IHH cells are also regions of frequent mutations in diseased human livers.

To determine whether there is an excess of SNVs at the TERT promoter in preneoplastic cirrhotic liver, we looked for mutations in the corresponding region of sequenced whole genomes from human donors [26]. However, this did not reveal an increased incidence of mutation in preneoplastic cirrhotic liver. In comparison, 10 out of 26 tumours (38.4%) had at least 3 reads in at least 1 sequenced microdissection supporting a TERT mutation (i.e. mutation in hg19: between Chr5, positions 1,295,228 and 1,295,250). This result could be explained by the TERT promoter DNA mutations being at such a low frequency in cirrhotic livers that much deeper sequencing of liver is needed (which may be expected considering the low frequency of hepatocyte transformation in cirrhotic liver). Alternatively, the TERT promoter region mutations may arise at some point between cirrhosis and the ‘initiated cell’ or ‘preneoplastic lesion’ stages.

To determine whether there is an excess of structural variations (SV) at oleic acid peak regions compared to control peaks, we re-examined the respective ChIP-seq peak regions in our sequenced whole genomes from human donors. While we did not observe any SVs at the control peak regions, we observed a deletion that affects *ARID5A*, *KANSL3* and *CNNM4*, and a translocation that affects *PTPRN2* in the oleic acid condition. It is intriguing that *KANSL3* is a glucose metabolism-driven master regulator of genome-wide histone H4 acetylation [40]. It is also prognostic for survival probability, with high expression unfavourable in liver cancer (The Human Protein Atlas). Additionally, *PTPRN2* has been identified as one of 4 regions with abnormal epigenetic state in HCC [41]. Therefore, future research should consider structural variations, genomic instability and single base mutations due to steatosis-associated epigenomic alterations.

Finally, we examined the mutational signatures that are present in the human liver biopsy WGS datasets at the regions which correspond to the ɣH2AX peak regions. This indicated that the proportion of SNVs which are attributable to ROS-associated mutational processes are more pronounced in NAFLD clones in the steatotic ɣH2AX peak regions than control ɣH2AX peak regions (Additional file 2: Fig S3). Indeed, the ROS-associated mutational signature had the greatest proportional increase in the ɣH2AX peak regions between healthy and NAFLD livers.

### The TERT gene has high levels of histone acetylation and ɣH2AX in steatosis which can be reversed with acetylation inhibitors

**Fig. 4:**
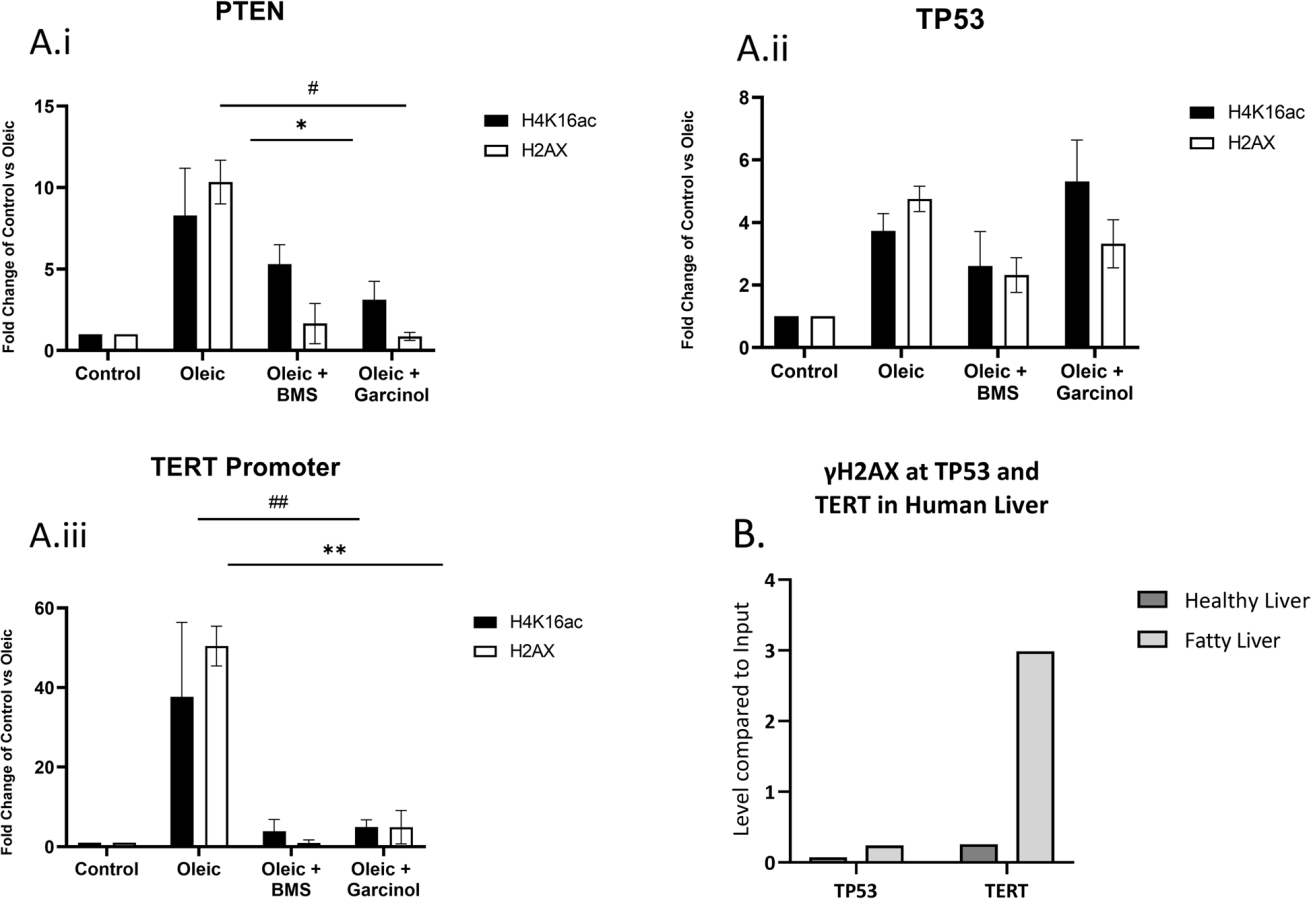
ChIP-qPCR analyses ɣH2AX and H4K16ac levels at *PTEN* (**Ai**), *TP53* (**Aii**) and *TERT* promoter (**Aiii**) in IHH cells cultured in control media, oleic acid or oleic acid plus inhibitor drugs. Error bars are ± SEM. All treatments *n* = 3. ^#,*^P < 0.05, ^###^*P* < 0.0001 in 1-way ANOVA, * H4K16ac and # ɣH2AX. **B** ChIP-qPCR analyses ɣH2AX at *TP53* and *TERT* promoter in 3 samples from one human healthy liver and one human fatty liver

Mutations at the *TERT* gene are the earliest detected in HCC [7, 8]. We validated the ChIP-seq results with ChIP-qPCR of the *TERT* promoter region compared to other tumour suppressor genes which are known to become mutated in later stages of hepatocellular carcinogenesis. This showed an approximate 40-fold increase in both H4K16ac and ɣH2AX at the *TERT* promoter in steatotic IHH cells compared to control cells (Fig. 4A). Near the *PTEN* gene, the increase in both histone modifications was more modest, while *TP53* was even less changed (Fig. 4A). Treatment of the cells for 4 h with the ACLY inhibitor BMS or histone acetyltransferase inhibitor garcinol prior to ChIP-qPCR significantly reversed the steatosis-associated elevation in both H4K16ac and ɣH2AX levels at the *TERT* promoter. These data also confirmed that locus-specific histone acetylation was altered by inhibition of acetyl-CoA biogenesis. This was valuable as quantitation of histone acetylation with immunofluorescence (similar to ɣH2AX, see Fig. 2) had not been possible due to the presence of H4K16 acetylation in all cell types.

Again, it was important to support these cell culture observations with human samples. We were able to confirm the regional differences in ɣH2AX levels at the *TERT* promoter and *TP53* in human livers which had been rejected for transplantation (Fig. 4B). A high level of ɣH2AX enrichment was only seen at *TERT* in fatty livers.

### Network modelling predicts metabolic consequences of oleic acid treatment and reversal by metabolic inhibitors

To gain mechanistic insight into how ɣH2AX levels are increased by oleic acid and decreased by drugs which inhibit acetyl-CoA production, we used genome-scale metabolic network modelling [32, 33]. We used the Recon1 metabolic model that contains the relationship between 3744 reactions, 2766 metabolites, 1496 metabolic genes and 2004 metabolic enzymes curated from literature [31]. To create a hepatocyte-specific metabolic model, gene expression data from AML12 hepatocyte cells [13] was overlaid onto the Recon1 network model (Methods). The model was further constrained using the nutrient conditions in culture and reaction fluxes were determined using an optimization approach that determines the metabolic flux state that satisfies constraints from gene expression, nutrient conditions, thermodynamics and stoichiometry [33].

**Fig. 5:**
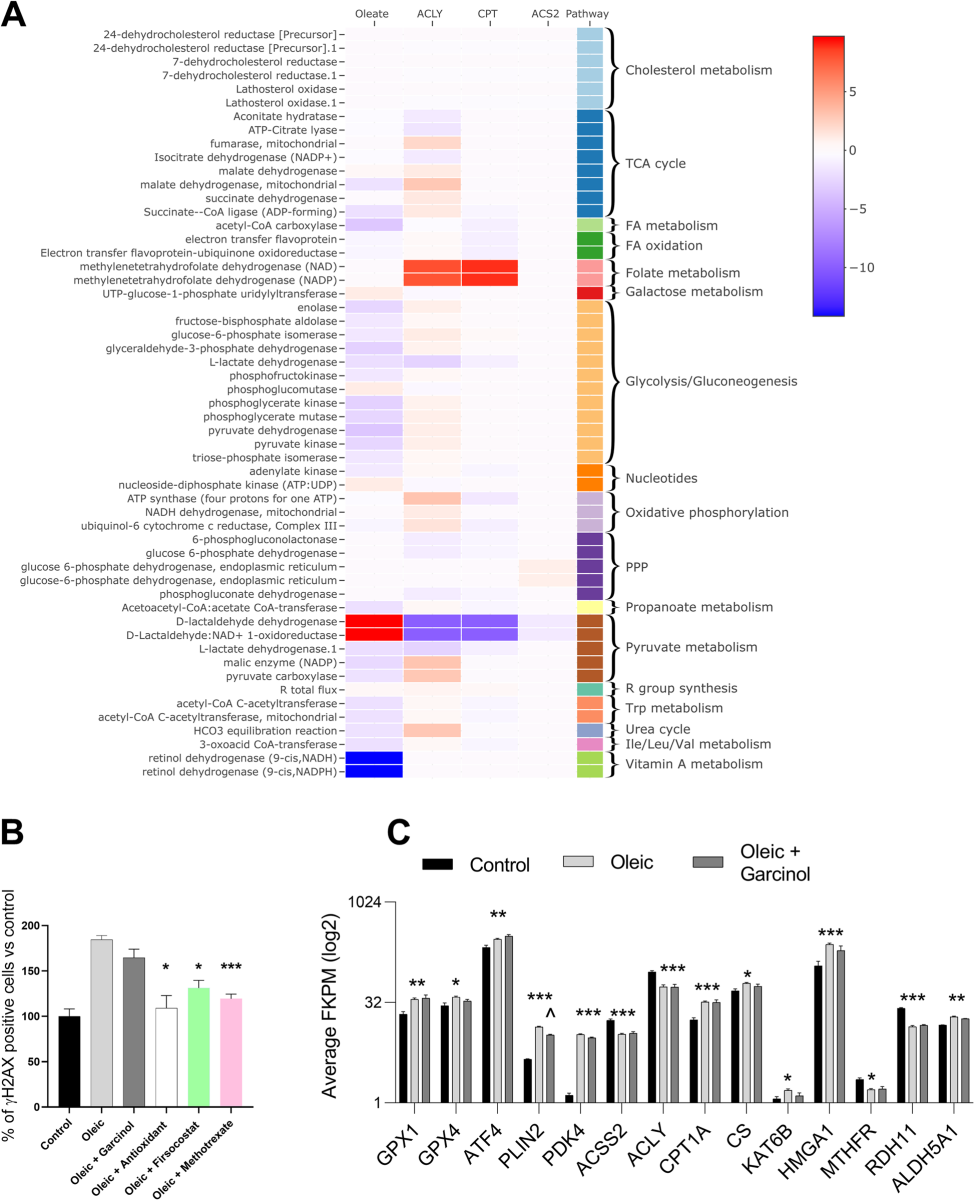
**A** Genome-scale metabolic modelling to simulate the impact of fatty acid treatment and metabolic enzyme inhibition on the metabolic network of hepatocytes. Heatmap of *z*-transformed reaction flux differences between the oleic acid vs control medium and ACLY, CPT, ACS2 KO vs wild type control in oleic acid-treated cells. *p* < 0.05 in any condition. **B** Average number of cells with high ɣH2AX after oleic acid or oleic acid plus drug treatments (% compared to control cells). Error bars are ± SEM. All groups *n* = 8. *P < 0.05, ***P < 0.0001 in *t*-test of oleic vs oleic plus inhibitor groups. **C** Select genes from the RNA-seq showing oleic acid-induced alteration to biological processes, oxidative stress response (GPX1, GPX4), lipotoxicity (ATF4), lipid droplet formation (PLIN2), acetyl-coA metabolism (PDK4, ACSS2, ACLY), β-oxidation (CPT1), the TCA cycle (CS), histone acetylation and chromatin condensation (KAT6B, HMGA1), folate metabolism (MTHFR), retinol dehydrogenase (RDH11) and aldehyde dehydrogenase (ALDH5A1). Garcinol treatment had little or no impact on any of these genes and processes. Error bars are ± SEM. All groups *n* = 4. *P < 0.05, **P < 0.001, ***P < 0.0001 in *t*-test of control vs oleic group, ^*P* < 0.05 in *t*-test of garcinol vs oleic group

This analysis suggested that oleic acid treatment had numerous metabolic effects, impacting central carbon, fatty acid, amino acid and folate metabolic pathways. The model predicts that both the steatosis-associated increase of ɣH2AX and its reversal is linked to redox metabolism. Many reactions which require the redox couples NAD+ /NADH or NADP+ /NADPH were altered in opposite directions in oleic acid treatment compared to the metabolic conditions induced by the inhibitors (Fig. 5A). Apart from the common alteration in redox metabolism, the three acetyl-CoA inhibitors (Fig. 2A) were predicted to have distinct impact on the metabolic network. Both ACLY and CPT1 inhibition were predicted to increase folate metabolism, whereas ACSS2 inhibition did not. CPT1 inhibition further reduced oxidative phosphorylation. We tested these predictions in our in vitro model by administering an antioxidant cocktail supplement or the folic acid analogue methotrexate to cells after oleic acid-induced steatosis. Both of these supplements significantly reduced the levels of ɣH2AX (Fig. 5B).

### Transcriptomic investigation of the in vitro model supports the importance of oxidative stress and reveals steatosis-associated increases in non-coding RNA

To further investigate the cellular processes altered by oleic acid supplementation and those which could underlie the changes to histone acetylation and ɣH2AX, we performed RNA-seq on IHH cells from 3 groups. The groups were cells grown in control media, cells grown in oleic acid-supplemented media and cells grown in oleic acid-supplemented media with a 4-h treatment with the histone acetyltransferase inhibitor garcinol. The previous assays had shown that these groups differed in the levels of both histone acetylation and ɣH2AX, with the control media having low levels of both, the oleic acid-supplemented group having high levels of both, and the oleic acid plus garcinol group having a reduction of both at the *TERT* locus (Fig. 4Aiii), but no reduction of global ɣH2AX (Fig. 5B).

Gene expression analysis identified 1769 upregulated and 2081 downregulated transcripts in oleic acid-supplemented IHH cells compared to those grown in control media (Additional file 2: Fig S4A – volcano plot). Gene Set Enrichment Analysis (Additional file 4: RNA-seq DEG and GSEA) revealed that 13 of the top 20 most significantly increased gene sets were related to chromatin regulation, and 2 of top 20 were linked to cell proliferation and mitosis. The most significantly downregulated processes were involved in biosynthesis of lipids, steroids and alcohol. Examination of select differentially expressed genes supported the expected oleic acid-induced increases in β-oxidation (*CPT1A*), lipid storage (*PLIN2*), lipotoxicity (*ATF4*) and oxidative stress responses (*GPX1*, *GPX4*) (Fig. 5C). Genes involved in acetyl-CoA biogenesis, in pathways other than β-oxidation were downregulated (*ACSS2*, *ACLY*, *PDK4*), and the computationally predicted increase in aldehyde dehydrogenase (*ALD5A1*) and decrease in retinol dehydrogenase were also observed.

Gene expression analysis identified 532 upregulated and 535 downregulated transcripts in garcinol plus oleic supplemented cells versus those which were supplemented with just oleic acid media (Additional file 2: Fig S4B – volcano plot). Gene Set Enrichment Analysis (Additional file 4: RNA-seq DEG and GSEA) revealed that cell and organelle developmental gene sets were common in both the top 20 significantly increased and top 20 significantly decreased lists. The transcription factor *JUN* was the most significantly downregulated transcript which may indicate that garcinol reduces cell proliferation or increases apoptosis. Additionally, there was no indication that β-oxidation (*CPT1A*), lipotoxicity (*ATF4*), acetyl-CoA biogenesis (*ACLY, ACSS2, PDK4*) nor oxidative stress responses (*GPX1, GPX4*) were altered by garcinol although a slight decrease in a marker for lipid storage was seen (*PLIN2*) (Fig. 5C).

We identified the genes which had reciprocal significant expression changes due to oleic acid supplementation compared to garcinol treatment in order to identify candidates that could explain both the increased and decreased ɣH2AX levels, respectively (Additional file 2: Fig S4 C, D – Venn diagrams). Gene Ontology analysis of these with DAVID identified 13 significantly enriched Biological Processes for genes which had increased expression in oleic acid but decreased by garcinol. 8/13 were related to transcriptional and translational regulation, but proliferation, apoptosis and telomerase activity were also identified (Additional file 4: RNA-seq DEG and GSEA).

We also used the RNA-seq data to investigate whether the epigenome change could be causing locus-specific or genome-wide transcriptional effects. We firstly examined the regions which were identified to have the largest H4K16 acetylation and ɣH2AX ChIP-seq peak increases in oleic acid-supplemented IHH cells compared to control cells (as described above, clustered on chromosomes 5, 7, 11, 15 and 19). There did not appear to be any overall cluster-specific transcriptional effect on protein-coding genes, with all clusters having a mix of significantly up- and downregulated genes. However, the RNA-seq also revealed that oleic acid-treated cells had a greater percentage of lincRNA or antisense transcripts with significantly increased expression (9.4% of all DE transcripts), than were significantly decreased (5.8% of all DE transcripts) which was a divergence from the relative percentage of all combined transcript types (45% up-, 54% downregulated). Closer examination of the ChIP-seq enriched gene clusters also revealed that the highest upregulated transcript in each cluster was a non-coding RNA. The effects of garcinol treatment may mirror this, as the most significantly downregulated transcripts in 3 of the 5 clusters were non-coding RNAs (the other 2 clusters had only significantly differentially expressed protein-coding genes).

## Discussion

In this study, we provide evidence that energy metabolism can direct epigenetic change and DNA damage across the genome, including to the earliest mutated gene in HCC. While acetylation changes and HDAC expression changes are known in HCC [20] and NAFLD [42], the relationship between histone acetylation and DNA mutations at known HCC driver genes has not been reported. Our focus on non-cancerous, pre-mutational hepatocytes provides insight into the priming steps of an increasingly common cancer which has a 5-year survival of 19% [43] and is responsible for more than 700,000 attributable deaths per year worldwide [44] and may explain why HCC risk increases in NAFLD patients from the early stages of steatosis. It is also, to our knowledge, the first evidence for abnormal histone acetylation being the initiation event in carcinogenesis.

Overall, our data support the concept that increased acetyl-CoA flux in steatotic hepatocytes leads to increased histone acetylation at certain genomic regions thus increasing their sensitivity to the prevailing oxidative stress. We observed that many of the genomic regions which had the highest increases in ɣH2AX and H4K16ac in steatosis also had relatively high levels (compared to the rest of the genome) in cells grown in control media. This suggests that these regions, such as the telomere-proximal cluster which contains *TERT*, may be predisposed for sensitivity [45]. There is an abundance of recent literature on the role of epigenetic modifications altering the expression of TERT in cancer via transcriptional and post-transcriptional mechanisms [46], but the potential for the epigenetic states promoting its mutagenesis has been hypothetical [47]. Despite the apparent risks for generating permanent mutations in pathologically important genes, the mutagenic metabolic environment can be rapidly reversed through alterations to energy and redox metabolism as shown with only 4 h of treatment with various inhibitors. Further investigation is needed to confirm that this can be done safely in vivo, in particular by inhibiting acetyl-CoA production, lipogenesis and/or histone acetylation in rodent models with severe diet-induced liver damage.

The computational modelling of the metabolic changes induced by the different inhibitors of acetyl-CoA production suggests that reduced oxidative stress is responsible for the ɣH2AX decreases. This is supported by the presence of ROS-associated mutational signatures of the SNVs that are found at the genomic regions in fatty livers which correspond to the oleic acid-induced ɣH2AX peaks. NAD/NADP-consuming enzymes in folate and pyruvate metabolism were predicted to be altered, and dysregulation of either process has been shown to influence oxidative stress in liver [48, 49]. The reversal of oleic acid-induced ɣH2AX in vitro by supplementing the cells with an antioxidant cocktail or the folic acid analogue methotrexate also supported the importance of these pathways. Telomeres are known to be particularly sensitive to damage due to oxidative stress [50], and further work is needed to determine whether subtelomeric regions also have particular sensitivity in steatotic hepatocytes. Additional mechanistic areas to investigate include further dissection of the relative importance of oxidative stress, folate and pyruvate metabolism for genome-wide versus locus-specific epigenome change and DNA damage, and seeing how other NAFLD-promoting lipid species such as palmitic acid influence the process. Moreover, in vivo and in vitro confirmation is required that inhibition of histone acetylation can prevent DNA mutations (as opposed to just reversing the indirect marker of DNA damage, ɣH2AX).

The RNA-seq comparison of control media and oleic acid supplementation in IHH cells suggested that the increase in histone acetylation and ɣH2AX are linked to increased oxidative stress due to lipotoxicity and/or increased ROS generation from mitochondrial fat metabolism. These data also highlighted the possibility that oleic acid stimulated cell proliferation may contribute to increases in genome-wide acetylation. Garcinol treatment provided a variety of mechanistic insights as it reverses histone acetylation and ɣH2AX at the *TERT* gene (Fig. 4Aiii) but not genome-wide (Fig. 5B). The treatment also did not appear to impact fat metabolism or oxidative stress (RNA-seq data). These results indicate, at least in vitro, that while inhibition of histone acetylation is sufficient to prevent DNA damage, it can only do so only at a subset of genomic regions.

The RNA-seq data also revealed that an in vitro transcriptomic consequence of epigenome change in steatosis is increased transcription from lowly expressed non-coding genes. This fits with the transcriptionally permissive nature of histone hyperacetylation which may stimulate transcription from cryptic promoters. It will be important to identify whether these transcripts contribute to pathogenic changes in hepatocytes either by themselves, perhaps by promoting structural and base pair-level mutability, or by modulating the expression of protein-coding genes.

The worldwide rates of HCC are increasing, and there is a switch from the incidence being driven by a relatively small population with viral hepatitis who have a high risk for HCC, to a large population with NAFLD who have a relatively low HCC risk [51]. Therefore, it is of interest to identify whether this new pathological mechanism is more pronounced in the small proportion of NAFLD patients who develop HCC. Our patient liver mutational analyses suggest that the same mechanism occurs in ARLD and T2D, which could potentially link increased HCC risk in the setting of steatohepatitis. Indeed, this mechanism may explain why in spite of differences in the mechanistic origin of hepatocyte lipid accumulation and oxidative stress [52], the same areas of the genome are mutated in preneoplastic liver and HCC [7, 26] in NAFLD and ARLD patients.

Besides HCC, future work will aim to determine whether regionally increased histone acetylation can progress the severity of NAFLD to NASH and cirrhosis through dysregulation of genes involved in inflammation and fibrosis. Finally, several drugs undergoing preclinical and clinical trials for reducing liver steatosis and fibrosis in NAFLD will likely impact acetyl-CoA metabolism [53] and/or histone acetylation [54–56]. Accordingly, we observed that firsocostat [57–59], an acetyl-CoA synthetase inhibitor which blocks the use of acetyl-CoA for DNL, reduces oleic acid-induced ɣH2AX in vitro (Fig. 5B). The clinical success of drugs which aim to reverse pathogenic liver metabolism in NAFLD/NASH has so far been modest [60]. However, our work indicates that the effects of these drugs on histone acetylation and DNA damage should also be evaluated in vivo.

## Conclusions

Our investigations reveal a new mechanism in which macronutrient-sourced metabolites induce transcriptional changes and promote mutations across the genome, including at oncogenes. The observation that the *TERT* gene is particularly affected indicates that this mechanism may be of particular importance for understanding the preneoplastic stages of HCC. To our knowledge, this is the first example of histone hyperacetylation being an initiating event in carcinogenesis. Future work can aim to manipulate this mechanism for reduction of HCC risk in patients with fatty liver disease.

## Supplementary Information

**Additional file 1.** Liver Cohort Baseline Clinical Data.

**Additional file 2.** Supplementary Figures S1-S4.

**Additional file 3.** ChIPseq significant histone peaks.

**Additional file 4.** RNAseq DEG and GSEA.

## Data Availability

The ChIP-seq and RNA-seq data generated and analysed in this study are available in the NCBI Short Reads Archive, BioProject accession number PRJNA741105 at http://www.ncbi.nlm.nih.gov/bioproject/741105 and can be downloaded from the SRA Run Selector https://trace.ncbi.nlm.nih.gov/Traces/study/?acc=PRJNA741105&o=acc_s%3Aa [23]. The human whole genome sequencing data is available at EGA https://ega-archive.org/datasets/EGAD00001006255 [26].

## Abbreviations

Acetyl-CoA: Acetyl coenzyme A
ACLY: ATP citrate lyase
ACSS2 / ACS2: Acetyl-CoA synthetase
ALDH5A1: Aldehyde dehydrogenase 5 family, member A1
ARID5A: AT-rich interactive domain-containing protein 1A
ARLD: Alcohol-related liver disease
ATF4: Activating transcription factor 4
ChIP-seq: Next-generation sequencing of chromatin immunoprecipitated DNA
CNNM4: Cyclin And CBS Domain Divalent Metal Cation Transport Mediator 4
CPT-1 / CPT: Carnitine palmitoyltransferase-1
CS: Citrate synthase
DNL: De novo lipogenesis
GPX1/4: Glutathione peroxidase1/4;
H4K16: Histone H4 lysine 16
HAT: Histone acetyltransferase
HCC: Hepatocellular carcinoma
HDAC: Histone deacetylase
HFD: High-fat diet
HMGA1: High-mobility group protein HMG-I/HMG-Y
IHH: Immortalized human hepatocytes
KANSL3: KAT8 regulatory NSL complex subunit 3
KAT6B: K(lysine) acetyltransferase 6B
MTHFR: Methylenetetrahydrofolate reductase
NAD+ /NADH: Nicotinamide adenine dinucleotide, oxidized and reduced forms, respectively
NADP+ /NADPH: Nicotinamide adenine dinucleotide phosphate, oxidized and reduced forms, respectively
NAFLD: Non-alcoholic fatty liver disease
PDK4: Pyruvate dehydrogenase kinase 4
PLIN2: Perilipin 2
PTPN2: Protein tyrosine phosphatase non-receptor type 2
RDH11: Retinol dehydrogenase 11
RNA-seq: Next-generation sequencing of the transcriptome
SNV: Single-nucleotide variant
T2D: Type 2 diabetes
TCA: Tricarboxylic acid
TERT: Telomerase gene
WGS: Whole genome sequencing
γH2AX: Phosphorylated histone variant H2AX
