# Correction: Acetyl-CoA metabolism drives epigenome change and contributes to carcinogenesis risk in fatty liver disease

**DOI:** 10.1186/s13073-023-01190-7

**Published:** 2023-05-18

**Authors:** Gabriella Assante, Sriram Chandrasekaran, Stanley Ng, Aikaterini Tourna, Carolina H. Chung, Kowsar A. Isse, Jasmine L. Banks, Ugo Soffientini, Celine Filippi, Anil Dhawan, Mo Liu, Steven G. Rozen, Matthew Hoare, Peter Campbell, J. William O. Ballard, Nigel Turner, Margaret J. Morris, Shilpa Chokshi, Neil A. Youngson

**Affiliations:** 1 Institute of Hepatology, Foundation for Liver Research, 111 Coldharbour Lane, London, SE5 9NT UK; 2 Faculty of Life Sciences and Medicine, King’s College London, London, UK; 3 Program in Chemical Biology, University of Michigan, Ann Arbor, MI 48109 USA; 4 Center for Bioinformatics and Computational Medicine, Ann Arbor, MI 48109 USA; 5 Department of Biomedical Engineering, University of Michigan, Ann Arbor, MI 48109 USA; 6 Rogel Cancer Center, University of Michigan Medical School, Ann Arbor, MI 48109 USA; 7 Wellcome Trust Sanger Institute, Cambridge, UK; 8 UNSW Sydney, Sydney, Australia; 9 Cellular Bioenergetics Laboratory, Victor Chang Cardiac Research Institute, Darlinghurst, NSW Australia; 10 Institute of Liver Studies, King’s College Hospital, London, UK; 11 Programme in Cancer and Stem Cell Biology, Duke-NUS Medical School, Singapore, Singapore; 12 CRUK Cambridge Institute, Cambridge, UK; 13 Department of Medicine, University of Cambridge, Addenbrooke’s Hospital, Cambridge, UK; 14 Department of Ecology, Environment and Evolution, La Trobe University, Bundoora, Melbourne, VIC 3086 Australia

**Correction: Genome Med 14, 67 (2022)**

**https://doi.org/10.1186/s13073-022-01071-5**

The original publication of this article [1] contained an incorrect funding acknowledgement. The incorrect and correct information is listed in this correction article. The original article has been updated.

**Incorrect**

Camille and Henry Dreyfus Foundation and R35 GM13779501 from the National Institutes of Health (NIH) USA to S. Chandrasekaran

**Correct**

Camille and Henry Dreyfus Foundation and startup funds from the University of Michigan to S. Chandrasekaran.
