# Supplementary material for: Acetyl-CoA metabolism drives epigenome change and contributes to carcinogenesis risk in fatty liver disease
Source: Genome Med. 2022 Jun 23;14:67. doi: 10.1186/s13073-022-01071-5 (PMC9219160; doi:10.1186/s13073-022-01071-5)
Supplement: Supplementary file 2 — Additional file 2. Supplementary Figures S1-S4. [file 13073_2022_1071_MOESM2_ESM.docx]

Assante et al., **Acetyl-CoA metabolism drives epigenome change and contributes to carcinogenesis risk in fatty liver disease**. Supplementary Figures.


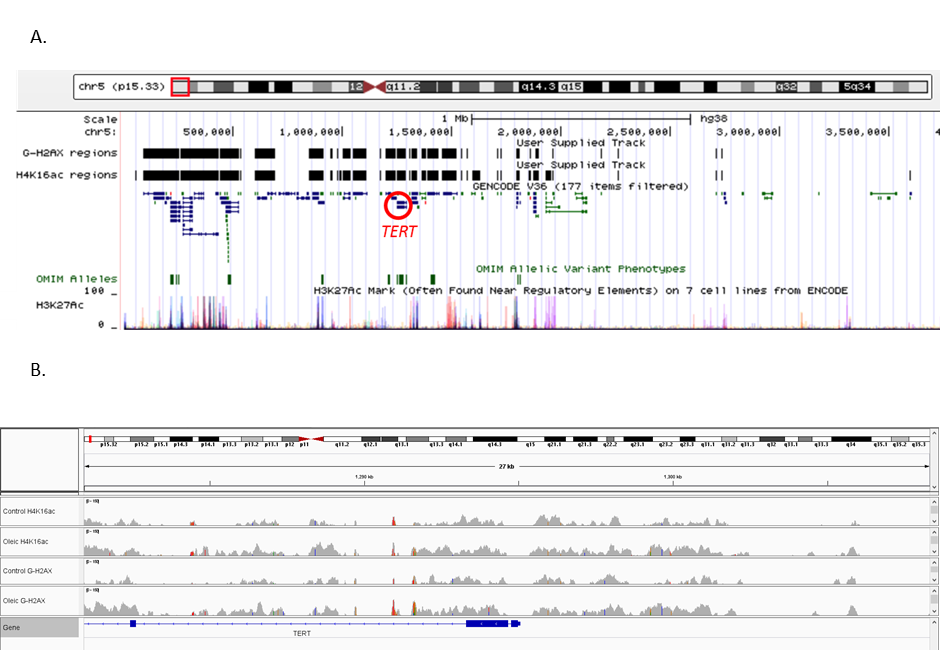


Supplementary Figure S1. A. UCSC Genome Browser of the chromosome 5 telomere-proximal gene cluster showing location of genes and ENCODE H3K27ac regions relative to ɣH2AX and H4K16ac ChIP-seq peak regions. B. IGV Genome Browser snapshot of the *TERT* promoter regions displaying aligned sequence reads from ChIP-seq files of the 4 samples top to bottom, control media cells H4K16ac, oleic acid treated cells H4K16ac, control media cells ɣH2AX, oleic acid treated cells ɣH2AX.


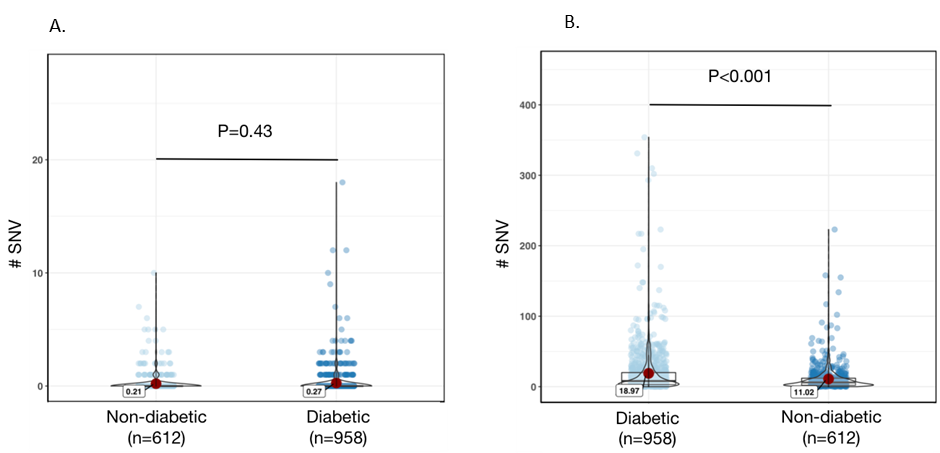


Supplementary Figure S2. Violin plots showing a comparison of the number of single nucleotide variants (SNVs) in individual genetic clones from donors with versus without diabetes, at A. control, and B. oleic acid peak regions. The median number of SNVs are annotated on the plots. Genetic clones from 20 non-diabetic patients, and 14 diabetics.


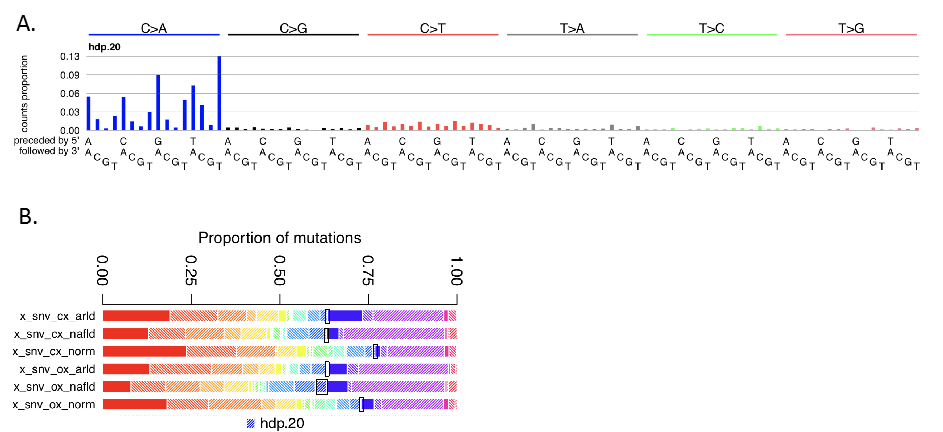


Supplementary Figure S3 A. A mutational signature (hdp.20) was detected with 97% cosine similarity to SBS18, indicating that a proportion of SNVs is attributable to a ROS associated mutational process and was most pronounced in NALFD clones at oleic acid peak regions. B. Bold black outlines highlight the relevant regions on the stacked bar plots corresponding to the ROS associated mutational signature. CX indicates control media cells ɣH2AX ChIP-seq peak regions, OX indicates oleic acid treated cells ɣH2AX ChIP-seq peak regions.


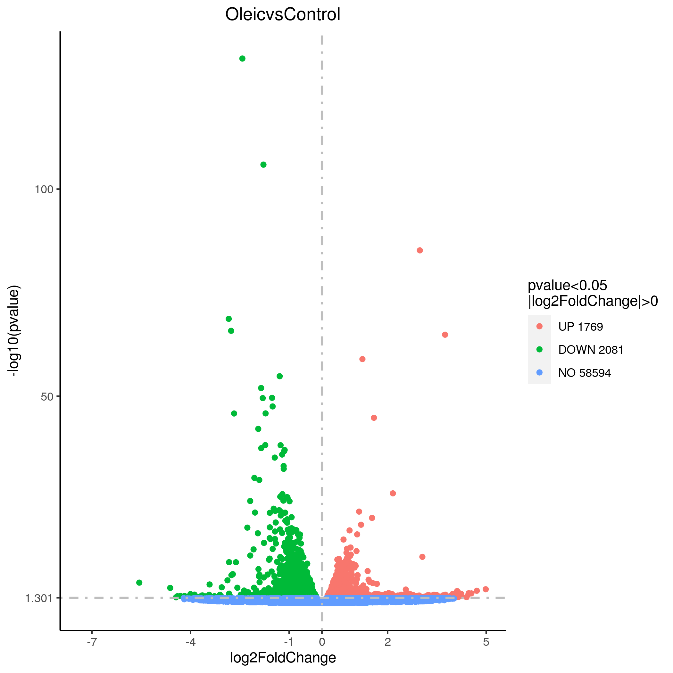

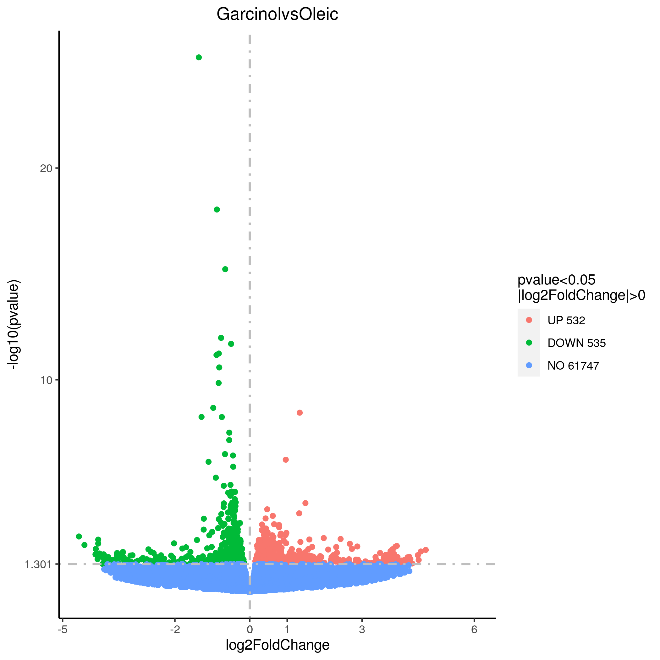


D.

C.

B.

A.

RHOB

TFRC

IFIT2

FOS

TXNIP

CYR61

JUN

LIPG

DHCR7

TXNIP

PLIN2

PDK4

ANGPTL4

SCDHCR7D

THBS1


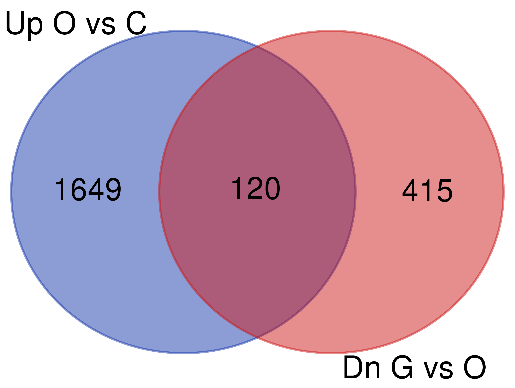

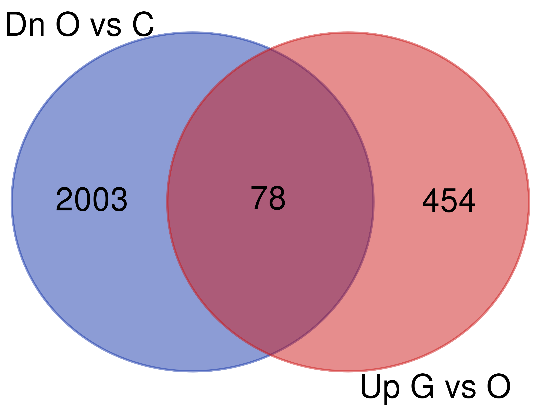


Supplementary Figure S4. A and B are volcano plots of IHH cell RNA-seq fold change and significance for differentially expressed genes. Transcripts with largest significance are indicated. A. Control versus Oleic acid treated cells, Up’ indicates genes with higher transcript levels in oleic acid treated cells. B. Oleic acid versus oleic acid plus Garcinol treated cells, Up’ indicates genes with higher transcript levels in garcinol plus oleic acid treated cells. C and D are venn diagrams comparing the significantly differentially expressed genes to reveal candidates for the reversal of epigenetic states. C. Genes upregulated in Oleic versus Control, and genes downregulated in Garcinol vs Oleic. The 120 common genes are potentially responsible for induction and reversal of epigenetic state. D. Genes downregulated in Oleic versus Control, and genes upregulated in Garcinol vs Oleic. The 78 common genes are also potentially responsible for induction and reversal of epigenetic changes.
